# TRPV-1-mediated elimination of residual iPS cells in bioengineered cardiac cell sheet tissues

**DOI:** 10.1038/srep21747

**Published:** 2016-02-18

**Authors:** Katsuhisa Matsuura, Hiroyoshi Seta, Yuji Haraguchi, Khaled Alsayegh, Hidekazu Sekine, Tatsuya Shimizu, Nobuhisa Hagiwara, Kenji Yamazaki, Teruo Okano

**Affiliations:** 1 Institute of Advanced Biomedical Engineering and Science, Tokyo Women’s Medical University, 8-1 Kawada-cho, Shinjuku, Tokyo, 162-8666, Japan; 2 Department of Cardiology, Tokyo Women’s Medical University, 8-1 Kawada-cho, Shinjuku, Tokyo, 162-8666, Japan; 3 Department of Cardiovascular Surgery, Tokyo Women’s Medical University, 8-1 Kawada-cho, Shinjuku, Tokyo, 162-8666, Japan

## Abstract

The development of a suitable strategy for eliminating remaining undifferentiated cells is indispensable for the use of human-induced pluripotent stem (iPS) cell-derived cells in regenerative medicine. Here, we show for the first time that TRPV-1 activation through transient culture at 42 °C in combination with agonists is a simple and useful strategy to eliminate iPS cells from bioengineered cardiac cell sheet tissues. When human iPS cells were cultured at 42 °C, almost all cells disappeared by 48 hours through apoptosis. However, iPS cell-derived cardiomyocytes and fibroblasts maintained transcriptional and protein expression levels, and cardiac cell sheets were fabricated after reducing the temperature. TRPV-1 expression in iPS cells was upregulated at 42 °C, and iPS cell death at 42 °C was TRPV-1-dependent. Furthermore, TRPV-1 activation through thermal or agonist treatment eliminated iPS cells in cardiac tissues for a final concentration of 0.4% iPS cell contamination. These findings suggest that the difference in tolerance to TRPV-1 activation between iPS cells and iPS cell-derived cardiac cells could be exploited to eliminate remaining iPS cells in bioengineered cell sheet tissues, which will further reduce the risk of tumour formation.

The fabrication of functional bioengineered tissues is a promising strategy for regenerative medicine. Various types of regenerative therapies that use tissue-engineering technologies have been applied to patients with impaired tissue/organ function^1^,^2^,^3^. Although the patient’s own somatic cells are used for the technology in some areas, the use of pluripotent stem cells, including induced pluripotent stem cells (iPS cells), will enable us to obtain a wide variety and quantity of cells, which might extend the range of application of regenerative medicine^4^. However, the risk of tumour formation, due to remaining undifferentiated iPS cells in fabricated tissues, remains to be resolved. Because billions of iPS cell-derived cells are expected to be used for transplantation in heart failure and diabetes, robust efforts will be necessary for more sensitive detection and effective, specific elimination of residual iPS cells in bioengineered tissues. Recently, Lin28 was reported to be a more sensitive marker gene for detecting iPS cells than Oct4 and Nanog^5^. Kuroda *et al*. showed that 0.1% Lin28 expression in iPS cells is equivalent to contamination of iPS cells at a rate of 0.1% in retinal pigment epithelial cells^5^. Although some recent studies have reported an iPS cell elimination strategy that uses methionine-free medium or human pluripotent stem cell specific lectin-toxin fusion protein^6^,^7^,^8^, to date no studies have measured the degree of elimination of residual iPS cells in tissues for regenerative medicine.

Transient receptor potential (TRP) channels represent a large number of nonselective cation channels that regulate calcium permeability and consist of multiple subfamilies, including the canonical (TRPC), melastatin (TRPM), ankyrin (TRPA), polycystic (TRPP), mucolipin (TRPML), and vanilloid (TRPV)^9^,^10^. These are activated by various types of stimuli, including shear stress, mechanical stretch, and temperature, and exhibit a variety of functions, including proliferation, differentiation, and cell death. TRPV-1, a subtype of TRPV, is activated at a high temperature (≥42 °C) in combination with chemical environmental stimuli, such as capsaicin^11^. Recently, TRPV-1 activation was reported to induce cell death or suppress tumour growth. Neural precursor cell-derived endovanilloids and Arvanil, a TRPV-1 agonist, induces cell death in high-grade astrocytoma via the TRPV-1-mediated activating transcription factor-3 endoplasmic reticulum stress pathway^12^. TRPV-1 also negatively regulates the epidermal growth factor receptor in intestinal epithelial cells and reduces the risk of neoplasia development^13^. Thus, TRPV-1 activation might be helpful to reduce the risk of tumour formation following iPS cell-derived cell transplantation. However, the effects of TRPV-1 activation on iPS cell proliferation and cell death remain unclear.

Conversely, TRPV-1 also has some functions on heart component cells, including cardiomyocytes, fibroblasts, and blood vessels. Heart muscle derived from TRPV-1 null mice impairs functional recovery following ischemic injury^14^. Although TRPV-1 expression and function in cardiac fibroblasts is unclear, TRPV-1 is expressed in human corneal fibroblasts and contributes to IL-6 release via mitogen-activated protein kinase signalling^15^. The functional effects of TRPV-1 might depend on the degree of TRPV-1 activation. Lower concentrations of capsaicin, a TRPV-1 agonist, induce endothelium-mediated vasorelaxation by producing nitric oxide, while higher concentrations induce vasoconstriction through smooth muscle cell contraction^16^. Nevertheless, it remains to be determined how TRPV-1 activation that is capable of inducing cell death in iPS cells affects iPS cell-derived cardiomyocytes and fibroblasts, as well as contributes to reduced contamination of residual iPS cells in bioengineered cardiac tissues.

The present study demonstrates for the first time that TRPV-1 activation through 42 °C culture and an agonist, N-oleoyldopamin (OLDA), induces cell death in human iPS cells, but not iPS cell-derived cardiomyocytes and fibroblasts. This further enables the fabrication of bioengineered cardiac tissues with less iPS cells. The negative effects of 42 °C culture on iPS cells correlated with upregulated TRPV-1 expression. Additionally, iPS cell death at 42 °C was TRPV-1-dependent. TRPV-1 expression in iPS cells at baseline and after 42 °C culture was greater than in cardiomyocytes, indicating a difference in tolerance to TRPV-1 activation, which could be exploited for the elimination of iPS cells.

## Results

### Cultivation at 42 °C is critical for human iPS cell survival

**Figure 1:**
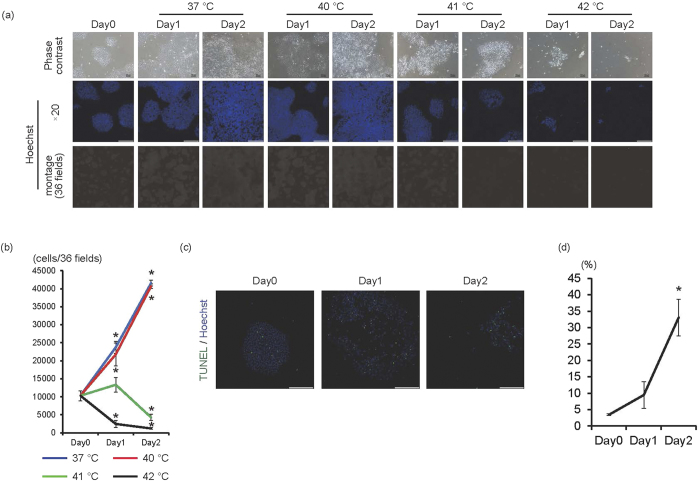
42 °C is the critical temperature for feeder-less human iPS cells. (**a**) Human iPS cells cultured on laminin E8 fragment were cultured at 37 °C, 40 °C, 41 °C, or 42 °C for 1 or 2 days. Upper panels are representative of phase-contrast images. Bars, 100 μm. Middle panels, Hoechst staining images. Bars, 200 μm. Lower panels, montage images of 36 fields (6 × 6) of Hoechst staining images (original magnification of each field is ×20). Multiple images from the same sample were acquired using the same microscope settings. (**b**) The cell number at each condition was calculated and shown in the graph (n = 3). *p < 0.01 vs. day 0. (**c**) Representative images of TUNEL staining (green). Nuclei were stained with Hoechst. (**d**) Percentage of TUNEL(+) cells was calculated and shown in the graph (n = 3). *p < 0.01 vs. day 0.

To determine whether high temperatures are harmful for iPS cells, feeder-free iPS cells were cultured at 37 °C, 40 °C, 41 °C, or 42 °C for 1 or 2 days (Fig. 1a,b). At 37 °C and 40 °C, cell number significantly increased in a time-dependent manner (*p* < 0.01, n = 3). Interestingly, cultivation at 41 °C induced cell growth arrest at day 1 and decreased cell numbers at day 2. However, cultivation at 42 °C significantly decreased cell numbers in a time-dependent manner (*p* < 0.01, n = 3). Accordingly, the number of Oct4-positive cells significantly increased at 37 °C (*p* < 0.01, n = 3), but significantly decreased at 42 °C (*p* < 0.01, n = 3) (Supplementary Fig. 1). Along with decreased cell numbers at 42 °C, the percentage of TUNEL (+) cells significantly increased in a time-dependent manner (*p* < 0.01, n = 3) (Fig. 1c,d), suggesting that 42 °C was the critical temperature for human iPS cell survival. Cytotoxic effects of 42 °C culture on feeder-less iPS cells were also observed in other type of iPS cells (1231A3 iPS cell line, Supplementary Fig. 2).

**Figure 2:**
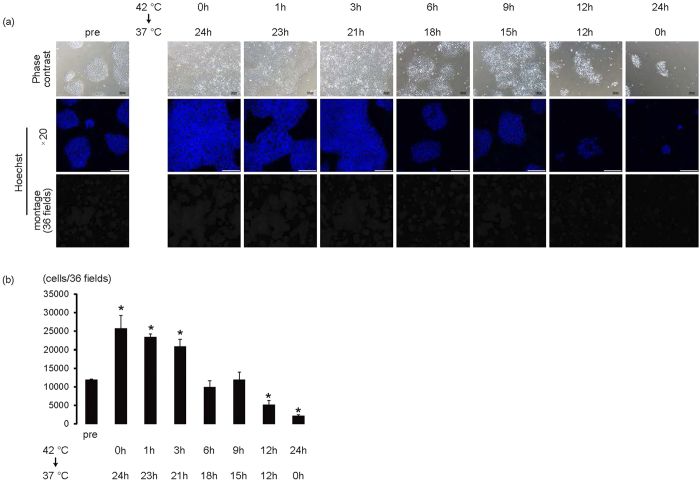
Over 12-hour culture at 42 °C is necessary for eliminating iPS cells. iPS cells on laminin E8 fragment were cultured for 1 hour, 3 hours, 6 hours, 9 hours, or 12 hours at 42 °C and subsequently cultured at 37 °C until 24 hours. (**a**) Upper panels are representative of phase-contrast images. Bars, 100 μm. Middle panels, Hoechst staining images. Bars, 200 μm. Lower, montage images of 36 fields (6 × 6) of Hoechst staining images (original magnification of each field is ×20). Multiple images from the same sample were acquired using the same microscope settings. (**b**) Cell number at each condition was calculated and shown in the graph (n = 3–4). *p < 0.01 vs. pre.

Next, we determine the minimum duration of 42 °C culture to eliminate iPS cells. When iPS cells were cultured at 42 °C for 1 or 3 hours, and subsequently cultured at 37 °C until 24 hours, cell numbers significantly increased (*p* < 0.01, n = 3–4); the increase in cell numbers was similar to the difference between the culture at 37 °C for 24 hours and day 0 (Fig. 2a,b). Although cell numbers remained unchanged in the 6- and 9-hour cultures at 42 °C, cultivation over 12 hours at 42 °C significantly decreased cell numbers compared with day 0 (*p* < 0.01, n = 3–4), suggesting that more than 12 hours at 42 °C might be necessary to eliminate iPS cells (Fig. 2a,b).

**Figure 3:**
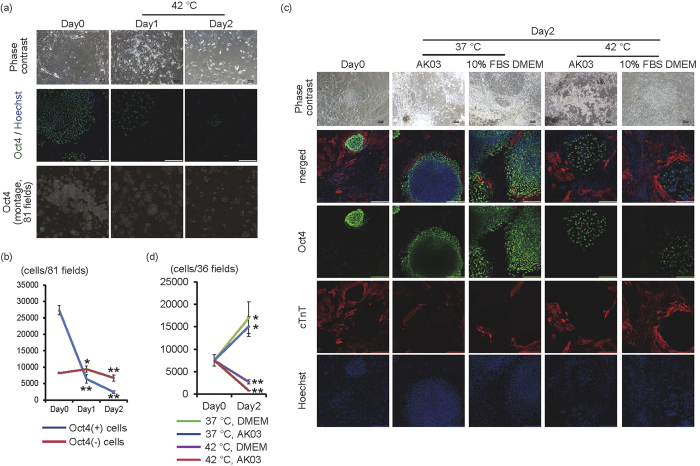
Effects of 42 °C culture on iPS cells in co-culture with other cells. (**a**) Human iPS cells cultured on MEF were cultured at 42 °C for 1 or 2 days. Upper panels are representative of phase-contrast images. Bars, 100 μm. Middle panels are representative images of Oct4 (green) and Hoechst (blue) staining. Bars, 200 μm. Lower, montage images of 81 fields (9 × 9) of Oct4 staining images (original magnification of each field is ×20). Multiple images from the same sample were acquired using the same microscope settings. (**b**) The Oct4(+) and Oct4(-) cell number in 81 fields at each time point was calculated and shown in the graph (n = 3). (**c**) Co-culture experiments between iPS cells and iPS cell-derived cardiac cells. Two days after starting co-culture of cell aggregates of iPS cells with iPS cell-derived cells, cells were cultured at 37 °C or 42 °C for 2 days in AK03 or 10% FBS DMEM. Upper panels are representative of phase-contrast images. Bars, 100 μm. Lower panels are representative of immunofluorescent images (Oct4; green, cTnT; red). Nuclei were stained with Hoechst (blue). Bars, 200 μm. (**d**) The Oct4(+) cell number in 36 fields at each time point was calculated and shown in the graph (n = 4). *p < 0.05 vs. day 0. **p < 0.01 vs. day 0.

Next, we examined whether 42 °C culture was also effective for eliminating iPS cells on mouse embryonic fibroblasts (MEF), because feeder cells might enhance iPS cell survival via mutual cell interactions. When iPS cells cultured on MEF were cultured at 42 °C, many of the cells that formed colonies disappeared by day 2, although there were still cells around the colonies (Fig. 3a). Confocal high-content image analysis revealed that the number of Oct4(+) cells significantly decreased in a time-dependent manner (*p* < 0.01, n = 3). On the other hand, the number of Oct4(−) cells significantly increased at day 1 (p < 0.05, n = 3) but decreased at day 2 (*p* < 0.01, n = 3) compared with day 0 (Fig. 3a,b). However many Oct4(−) cells still remained until day 2. These findings suggested that 42 °C might be toxic for iPS cells, but not for differentiated cells, such as feeder cells. Because cell-cell interaction might be different between cell species, it is important to determine whether iPS cells are eliminated at 42 °C in bioengineered tissues. Therefore, we evaluated whether iPS cells would survive or undergo cell death in co-culture with iPS cell-derived cardiac cells in AK03 or 10% FBS/DMEM, respectively. By using AK03 which is the suitable culture medium for iPS cells, it was possible to determine that iPS cells underwent cell death, even though they were cultured in AK03 with iPS cell-derived cardiac cells. Nevertheless, it is well known that cell-cell interaction depends on cell viability. Therefore, we evaluated iPS cell death in co-cultures with iPS cell-derived cardiac cells in 10% FBS/DMEM, which is the suitable culture medium for iPS cell-derived cardiac cells. When cell aggregates of iPS cells were co-cultured for 1 day with cardiac cells derived from human iPS cells, the iPS cells were engrafted with cardiomyocytes (Day0 in Fig. 3c,d). When these preparations were cultured for another 2 days at 37 °C, the iPS cells underwent significant proliferation in the iPS cell culture medium (AK03) or the medium for cardiomyocytes (10% FBS/DMEM), which led to increased colony size and displaced cardiomyocytes to the periphery of the colonies (Day2 in Fig. 3c,d). Conversely, when these preparations were cultured at 42 °C for 2 days, the number of Oct4(+) cells decreased, and iPS cells were sparse in the colonies (Day2 in Fig. 3c,d). These findings suggested that iPS cells might also be eliminated at 42 °C in co-culture conditions with various types of human iPS cell-derived differentiated cells.

### Cultivation at 42 °C is not critical for survival of human iPS cell-derived cardiomyocytes and non-cardiomyocytes

**Figure 4:**
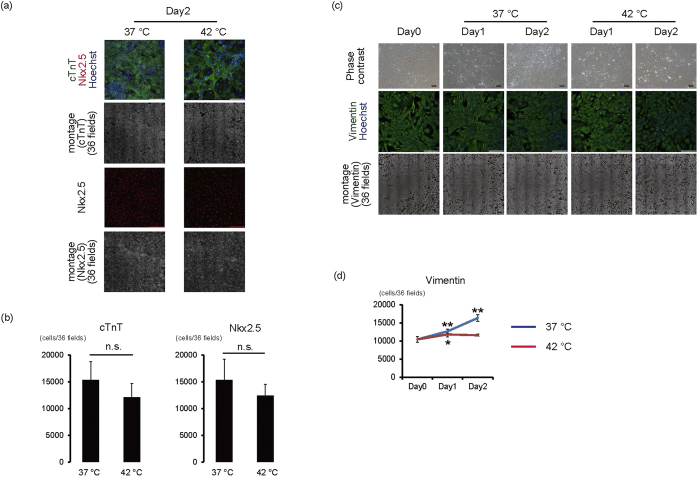
Effects of 42 °C culture on iPS cell-derived cardiomyocytes and fibroblasts. (**a**,**b**) iPS cell-derived purified cardiomyocytes were cultured at 37 °C or 42 °C for 2 days. (**a**) Representative images of cardiomyocytes. 1^st^ lines, merged images of Nkx2.5 (red), cTnT (green), and Hoechst (blue). 2^nd^ lines, montage images of 36 fields (6 × 6) of cTnT images (original magnification of each field is ×20). 3^rd^ lines, Nkx2.5 staining. 4^th^ lines, montage images of 36 fields (6 × 6) of Nkx2.5 images (original magnification of each field is ×20). Bars, 200 μm. Multiple images on cTnT and Nkx2.5 from the same sample were acquired using the same microscope settings. (**b**) The number of cTnT(+) and Nkx2.5(+) cells (n = 4) n.s., not significant. (**c**,**d**) iPS cell-derived fibroblasts after cardiac differentiation were cultured at 37 °C or 42 °C for 1 or 2 days. (**c**) Representative images of fibroblasts. 1^st^ lines, phase-contrast images. Bars, 100 μm. 2^nd^ lines, vimentin staining. Nuclei were stained with Hoechst. Bars, 200 μm. 3^rd^ lines, montage images of 36 fields (6 × 6) of vimentin images (original magnification of each field is ×20). Multiple images on vimentin from the same sample were acquired using the same microscope settings. (**d**) Number of vimentin(+) cells (n = 4). *p < 0.05 vs. day 0. **p < 0.01 vs. day 0.

**Figure 5:**
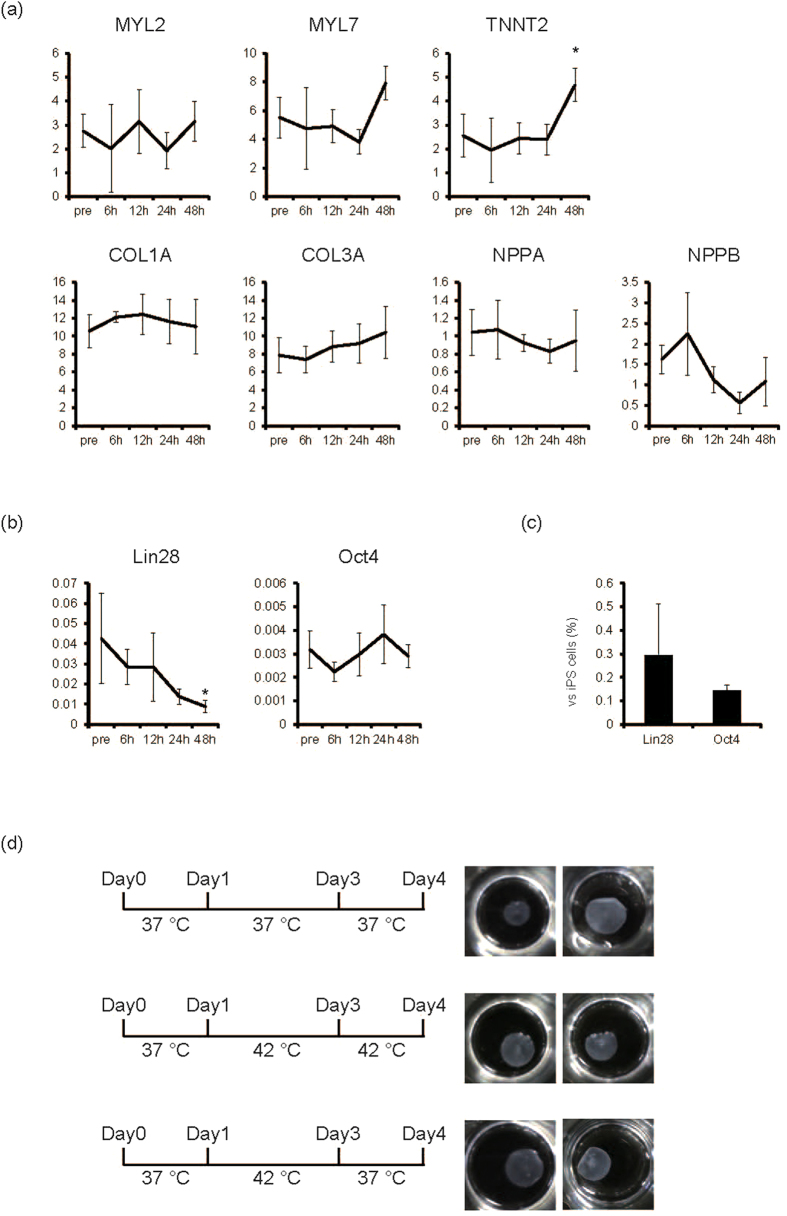
Elimination of remaining iPS cells in cardiac cell sheet preparation. (**a**) The mRNA expression in cardiac cells after cardiac differentiation of human iPS cells from each time point at 42 °C (n = 3). Y-axis indicates relative gene expression compared with GAPDH. *p < 0.05 vs. pre. (**b**) The mRNA expression of Lin28 and Oct4 of cardiac cells after cardiac differentiation of human iPS cells from each time point at 42 °C (n = 3). Y-axis indicates relative gene expression compared with GAPDH. *p < 0.05 vs. pre. (**c**) The relative mRNA expression of Lin28 and Oct4 of iPS cell-derived cardiac cells at 48 hours in 42 °C (n = 3, iPS cell = 100). Y-axis indicates relative gene expression compared with undifferentiated iPS cells cultured on MEF. (**d**) Effects of 42 °C culture on cell sheet fabrication. Four days after starting culture of iPS-derived cardiac cells in temperature-responsive multi-well culture plates at 37 °C, monolayered cell sheets were fabricated following lowering of culture temperature (upper). Even if cells were cultured at 42 °C from day 1 to day 4 (middle), or from day 1 to day 3 (lower), cell sheets were fabricated following lowering of culture temperature at day 4. Left, culture scheme. Right, macroscopic view of monolayered cell sheets from the different experiments.

It is necessary to determine the influence of 42 °C culture on differentiated cells for bioengineered tissues for this iPS cell elimination strategy to be successful and effective for regenerative medicine. Therefore, we evaluated the influence of 42 °C culture on iPS cell-derived cardiomyocytes and fibroblasts, the main cellular components of cardiac cell sheet tissues. When iPS cell-derived cardiomyocytes were cultured at 37 °C or 42 °C, cardiomyocytes exhibited spontaneous beating and many cells remained in both conditions until day 2 (Supplementary Video 1,2,3,4). This is consistent with the observation that the number of dead cardiomyocytes was not increased even after the cultivation at 42 °C (Supplementary Fig. 3a). Additionally, the number of cTnT- and Nkx2.5-positive cardiomyocytes was similar between the 37 °C and 42 °C condition on day 2 (Fig. 4a,b). Furthermore cardiomyocytes exhibited the spontaneous beating 1 week after the 2-day culture at 42 °C (Supplementary Video 5, 6). These findings suggest that cardiomyocyte cell survival might be unaffected by 42 °C until 2 days. Next we examined the effects of 42 °C culture on iPS cell-derived fibroblasts. When iPS cell-derived fibroblasts were cultured at 37 °C, the number of vimentin-positive iPS cell-derived fibroblasts significantly increased compared with day 0 (*p* < 0.01, n = 4) (Fig. 4c,d). Conversely, when iPS cell-derived fibroblasts were cultured at 42 °C, cell number significantly increased at day 1 (*p* < 0.05, n = 4) (Fig. 4c,d), but not at day 2 (Fig. 4c,d), suggesting that iPS cell-derived fibroblasts might be resistant to cell death at 42 °C. To determine molecular influences of 42 °C culture on iPS cell-derived cardiomyocytes and fibroblasts, we analysed mRNA expression of cardiac and extracellular matrix genes. When iPS cell-derived cardiac cells, including cardiomyocytes and fibroblasts, were cultured at 42 °C, mRNA expression of MYL2, MYL7, Col1A, Col3A, NPPA, and NPPB remained unchanged up to 48 hours. However, TNNT2 expression significantly increased at 48 hours (*p* < 0.05, n = 3) (Fig. 5a). These results suggested that transcriptional activity was maintained in 42 °C culture. Conversely, expression of Lin28, but not Oct3/4, significantly decreased in iPS cell-derived cardiac cells after cultivation at 42 °C (*p* < 0.05, n = 3) (Fig. 5b); Lin28 expression in cells cultured at 42 °C for 48 hours was 0.3% of that in iPS cells on MEF (Fig. 5c). Furthermore Lin28-positive cells were decreased to around 0.1% by 2-day culture at 42 °C (p < 0.05, n = 4) (Supplementary Fig. 4). These findings indicate that 42 °C culture might be useful for eliminating remaining iPS cells in iPS cell-derived cardiac cells.

Although there were minute effects of 42 °C culture on iPS cell-derived cardiac cells, it is important to evaluate the influence of 42 °C culture on the establishment of bioengineered cardiac tissues for use in regenerative medicine. As we previously reported, iPS cell-derived cardiac cells following cardiac differentiation were first cultured on temperature-responsive culture dishes for 4 days at 37 °C; results showed spontaneous beating of cardiomyocytes (Supplementary Video 7) and fabrication of monolayered cell sheets after lowering the culture temperature to 20 °C (Fig. 5d). Interestingly when iPS cell-derived cardiac cells were transiently cultured at 42 °C for 2 days (day 1 to day 3) or 3 days (day 1 to day 4), cell sheets were obtained after lowering the culture temperature (Fig. 5d), suggesting that elements needed to fabricate the bioengineered tissue, including cell-cell junction proteins, basement membrane proteins, and extracellular matrices, might be maintained to some extent. However, 3-day culture at 42 °C (day 1 to day 4) occasionally weakened cardiomyocyte beating (data not shown), while 2-day culture at 42 °C (day 1 to day 3) did not affect cardiomyocyte beating (Supplementary Video 8). As shown in Supplementary Fig. 3b, the number of dead cells in cardiac cell sheet component cells including cardiomyocyte and fibroblasts was not different between the 37 °C and 42 °C condition on day 1 and day 2 (Supplementary Fig. 3b). These findings suggest that 2-day culture at 42 °C during the process of cardiac cell sheet fabrication might be the optimal condition for eliminating remaining iPS cells with less affecting cardiomyocyte viability. Next we evaluated the function of cardiac cell sheets *in vivo*. When 2-layered cardiac cell sheets that were cultured at 37 °C or 42 °C for 2 days (Fig. 5d) were transplanted onto the subcutaneous tissue of nude rats, the transplanted cardiac cell sheets from both conditions showed the spontaneous beating at 1 week (Supplementary Video 9, 10). These findings suggest that transient culturing of iPS-derived cardiac cells at 42 °C during the fabrication of cell sheet preserves the contractility of the bioengineered tissues, and demonstrates the feasibility of this strategy in regenerative medicine.

### TRPV-1-mediated elimination of remaining iPS cells in human cardiac tissues

**Figure 6:**
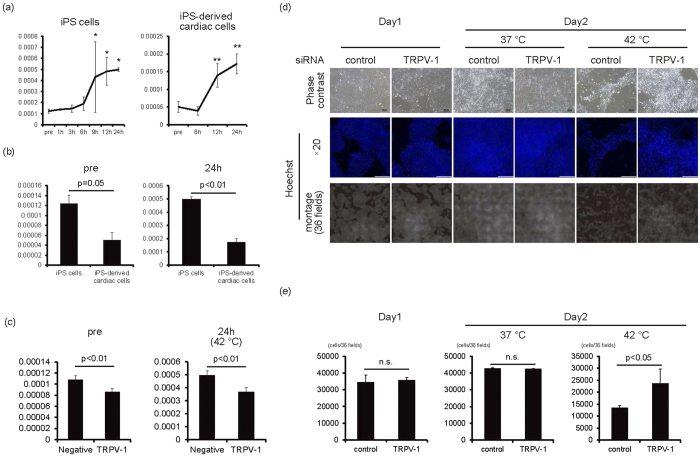
TRPV-1-mediated iPS cell elimination in 42 °C culture. (**a**) The mRNA expression of TRPV-1 on feeder-less iPS cells (left, n = 3) and iPS-derived cardiac cells (right, n = 3). Y-axis indicates relative gene expression of TRPV-1 compared with β-actin. *p < 0.05 vs. pre. **p < 0.01 vs. pre. (**b**) Comparison of TRPV-1 mRNA expression between feeder-less iPS cells and iPS-derived cardiac cells at pre and 24 hours in 42 °C culture (n = 3). (**c**) The mRNA expression of TRPV-1 in iPS cells transfected with TRPV-1 siRNA or control siRNA (n = 4). (**d**) One day after transfection of TRPV-1 siRNA or control siRNA (day 1), iPS cells were cultured at 37 °C or 42 °C for 1 day (day 2). Upper panels are representative of phase-contrast images. Bars, 100 μm. Middle panels, Hoechst staining images. Bars, 200 μm. Lower panels, montage images of 36 fields (6 × 6) of Hoechst staining images (original magnification of each field is ×20). Multiple images from the same sample were acquired using the same microscope settings. (**e**) Cell number at each condition was calculated and shown in the graph (n = 3).

It is well known that high temperature activates TRPV-1 and induces cytotoxic effects. We next analysed TRPV-1 expression in iPS cells and iPS cell-derived cardiac cells at 42 °C. TRPV-1 mRNA expression in iPS cells significantly increased after 9 hours at 42 °C (*p* < 0.05, n = 3) and in iPS cell-derived cardiac cells after 12 hours at 42 °C (*p* < 0.01, n = 3) (Fig. 6a). Conversely, TRPV-1 expression was significantly higher in iPS cells than in iPS-derived cardiac cells at baseline and after 24 hours at 42 °C (*p* < 0.05 for pre n = 3, *p* < 0.01 for 24 h, n = 3) (Fig. 6b). These findings suggested that a difference in TRPV-1 activation could account for varying effects of 42 °C culture between iPS cells and iPS cell-derived cardiac cells. To confirm the role of TRPV-1 in the cytotoxic effects of 42 °C culture on iPS cells, iPS cells were transfected with TRPV-1 siRNA. As expected, following TRPV-1 knockdown, the increase of TRPV-1 levels was lower than cells that were transfected with control siRNA (*p* < 0.01, n = 4) (Fig. 6c). Furthermore, the decrease in cell number following 42 °C incubation was prevented in cells transfected with TRPV-1 siRNA when compared to cells transfected with the control siRNA (Fig. 6d,e). These findings indicated that TRPV-1 activation might be critical for eliminating iPS cells in 42 °C culture.

**Figure 7:**
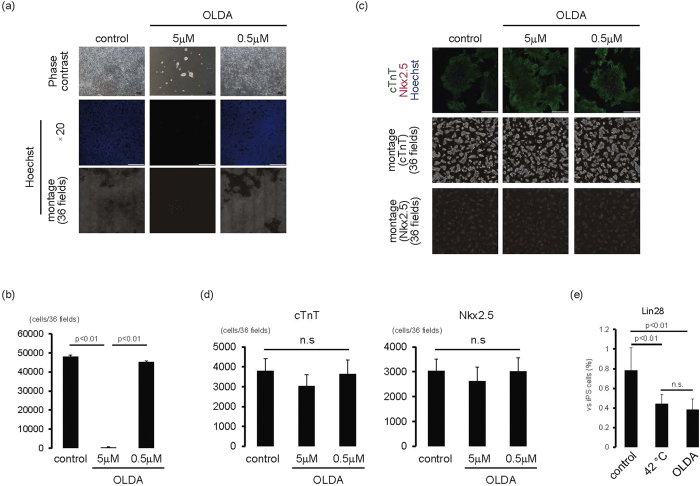
iPS elimination using TRPV-1 agonist. (**a**,**b**) iPS cells on laminin E8 fragment were cultured with OLDA for 1 day. (**a**) Upper panels are representative of phase-contrast images. Bars, 100 μm. Middle panels, Hoechst staining images. Bars, 200 μm. Lower, montage images of 36 fields (6 × 6) of Hoechst staining images (original magnification of each field is ×20). Multiple images from the same sample were acquired using the same microscope settings. (**b**) Cell number at each condition was calculated and shown in the graph (n = 3). (**c**,**d**) iPS cell-derived cardiac cells were cultured with OLDA for 1 day. (**c**) Upper, merged images of cTnT (green), Nkx2.5 (red), and Hoechst (blue). Bars, 200 μm. Middle, montage images of 36 fields (6 × 6) of cTnT staining images (original magnification of each field is ×20). Lower, montage images of 36 fields (6 × 6) of Nkx2.5 staining images (original magnification of each field is ×20). Multiple images on cTnT and Nkx2.5 from the same sample were acquired using the same microscope settings. (**d**) Number of cTnT-positive (left) and Nkx2.5-positive (right) cells. n.s., not significant. (**e**) iPS cell-derived cardiac cells were cultured at 42 °C or with OLDA (5 μM) at 37 °C for 2 days. The mRNA expression of Lin28 of cardiac cells after cardiac differentiation of human iPS cells (n = 3). Y-axis indicates relative gene expression of Lin28 compared with undifferentiated iPS cells cultured on MEF.

We next analysed whether pharmacological activation of TRPV-1 also induced toxic effects on iPS cells, but not cardiac cells. When feeder-less iPS cells were cultured with TRPV-1 agonists, including N-oleoyldopamine (OLDA) (Fig. 7a) and Arvanil (Supplementary Fig. 5), the number of cells significantly decreased in a dose-dependent manner (*p* < 0.01, n = 3) (Fig. 7a,b, Supplementary Fig. 5). Conversely, when iPS-derived cardiomyocytes were cultured with OLDA, spontaneous beating of cardiomyocytes continued (Supplementary Video 11,12,13) and the number of cTnT(+) and Nkx2.5 (+) cells was similar to cells cultured in vehicle (Fig. 7c,d). These findings suggested that TRPV-1 agonists might be useful for eliminating iPS cells with less affecting cardiomyocyte viability.

Finally, we examined how TRPV-1 activation treatments diminished the risk of iPS cell contamination in cardiac cells by analysing Lin28 expression. As shown in Fig. 7e, cultivation at 42 °C or with OLDA treatment for 2 days significantly decreased Lin28 expression to 0.4% compared with iPS cells (*p* < 0.01, n = 3).

## Discussion

The present study showed for the first time that TRPV-1 activation through the combination of culture at 42 °C and agonists induced cell death in human iPS cells, but not iPS cell-derived cardiomyocytes and fibroblasts. This allowed the fabrication of bioengineered cardiac cell sheet tissues. TRPV-1 expression in iPS cells at baseline and after 42 °C culture was higher than in cardiomyocytes; 42 °C culture-mediated iPS cell death was also TRPV-1-dependent.

High temperature is a stress for the living body. However, differences in heat tolerance between cells, as well as the threshold for heat-mediated cytotoxicity in human iPS cells, have not been fully elucidated. As shown in Fig. 1, 42 °C, but not 40 °C or 41 °C, is the critical temperature for human iPS cell survival when cultured in feeder-less conditions or in feeder conditions. Interestingly, negative effects on iPS cells at 42 °C were observed by 6 hours; cell cycle arrest and cell death was observed by 12 hours, suggesting that high-temperature sensing mechanisms might be regulated by high temperature itself. Although the regulatory mechanisms of TRPV-1 expression at high temperature are not fully understood, our results showed that TRPV-1 mRNA expression increases in a time-dependent manner at 42 °C. Additionally, expression is significantly upregulated by 9 hours compared with before cultivation at 42 °C (Fig. 6a), indicating that some degree of TRPV-1 expression might be necessary for cell death of human iPS cells. TRPV-1 is expressed in various types of cancers and capsaicin-induced cell death of MCF-7 breast cancer cell line has been reported to be dependent on TRPV-1 expression levels^17^. Although the transfection of TRPV-1 siRNA reduced the TRPV-1 expression only about 20% (Fig. 6c), the 42 °C culture-mediated cell death of iPS cells was inhibited, suggesting that the levels of TRPV-1 expression might be decreased below the threshold levels required its cytotoxicity in iPS cells. Conversely, TRPV-1 expression in iPS-derived cardiac cells at baseline and after 24 hours at 42 °C was significantly less than in iPS cells (Fig. 6b). Herein the difference in TRPV-1 expression between iPS cells and iPS cell-derived cardiac cells might be the reason for the differences of cytotoxicity at high temperature. Decreased TRPV-1 expression in iPS cell-derived cardiac cells might not be sufficient for the negligible effects of 42 °C culture on iPS cell-derived cardiac cells. Heating and capsaicin have been shown to activate TRPV-1 by shifting the voltage dependence of activation^18^; subsequent increased intracellular Ca^2+^ concentration leads to various biological functions, including hypertrophy^19^, proliferation^20^, cell growth inhibition^13^, and cell death^12^. Although we have not elucidated the membrane potentials of TRPV-1, the TRPV-1 channel on the membrane of iPS cells is also expected to open as a result of 42 °C culture and chemical compound treatment. However, because TPPV-1 expression in iPS cells is low at baseline, and the appearance of cell growth arrest or cell death at 42 °C correlates with increased TRPV-1 expression (Figs 2 and 6a), the upregulation of TRPV-1 might be necessary for heat and chemical compound-mediated cell death.

In the present study, the cultivation at 42 °C induced the apoptosis of iPS cells. Although the precise mechanisms on it remain unclear, some reports on TRPV-1-mediated apoptosis might give us some cues. The stimulation of TRPV-1 induced apoptosis of high-grade astrocytoma through the increase of activating transcription factor-3 expression and activating endoplasmic reticulum stress pathway^12^, and induced calcium entry-mediated reactive oxygen species production and mitochondria depolarization in rat synovial fibroblasts^21^. Furthermore the expression of adhesion proteins such as ICAM-1 and VCAM-1 have been reported to be upregulated in kidney of TRPV-1 null mutant mice compared with that of wild type mice in lipopolysaccharide intraperitoneal injection renal inflammation models, suggesting that TRPV-1 might contribute to cell death through affecting adhesion proteins expression^22^. On the other hand, cell-cell interaction in tissues might, to some extent, ameliorate TRPV-1-mediated cells death. As shown in Fig. 1 and Supplementary Figs 1 and 2, the cultivation at 42 °C induced the cell death in almost all of feeder-free iPS cells, while small number of iPS cells remained when co-cultured with MEFs or iPS cell-derived cardiac cells including fibroblasts (Fig. 3). Fibroblasts have been reported to secrete various types of extracellular matrix proteins including laminins and fibronectin, and have some influence on proliferation and cell death of surrounding cells through integrin signals. Human embryonic stem cells have been reported to express various types of integrin^23^. Although the growth and survival of feeder-free iPS cells cultured on laminin E8 fragment is mainly regulated by α6β1 integrin^24^, many types of integrin signal are expected to be activated in co-culture condition. Therefore iPS cells in bioengineered tissues might be protected against TRPV-1-mediated cell death and the optimal culture period at 42 °C or the optimal doses of TRPV-1 agonists might be necessary to eliminate residual iPS cells in bioengineered tissues.

A better understanding of the mechanisms of the cell death of iPS cells enables us to develop further strategies to eliminate iPS cells in bioengineered tissue. Because increased TRPV-1 expression plays a role in the death of iPS cells exposed to 42 °C, treatment of TRPV-1 agonists is thought to be an alternative strategy for eliminating iPS cells with less affecting cardiac cell viability. In the present study, treatment with 5 μM OLDA induced cell death in almost all iPS cells (Fig. 7a,b). However, the same OLDA dose did not affect spontaneous beating of cardiomyocytes or the number of surviving cardiomyocytes (Fig. 7c,d). Conversely, 50 mg/mL Arvanil induced cell death in almost all iPS cells (Supplementary Fig. 5), but also induced cell death in cardiomyocytes (data not shown). Although the mechanisms responsible for the varying effects of TRPV-1 agonists on cardiomyocytes remain unclear, a recent report suggested that OLDA attenuates ischemia/reperfusion injury of hearts via TRPV-1 activation^25^. Therefore, treatment with a suitable TRPV-1 agonist might eliminate iPS cells with less affecting cardiomyocyte viability.

TRPV-1 has been reported to have some functions on cardiovascular cells^26^. Heart muscle derived from TRPV-1 null mice exhibits impaired functional recovery after ischemic injury^14^, and TRPV-1 activation by 12-lipoxygenase-derived eicosanoids protects heart tissues against myocardial ischemia/reperfusion injury^27^. Conversely, pressure overload-mediated cardiac hypertrophy is inhibited in mice lacking TRPV-1^28^, and treatment with a TRPV-1 antagonist prevents cardiac dysfunction in a cardiac hypertrophy model^29^. Although the mechanisms of TRPV-1 activation on cardiomyocytes remain unclear, it is likely that the degree of TRPV-1 activation contributes to vascular function. Treatment with lower concentrations of capsaicin, a TRPV-1 agonist, induces endothelium-mediated vasorelaxation by producing nitric oxide, while higher concentration induces vasoconstriction through smooth muscle cell contraction^16^. In our present study, 2-day culture at 42 °C did not affect spontaneous beating of cardiomyocytes, while 3-day culture at 42 °C weakened spontaneous beating in some preparations. Consistent with reports that excess levels of intracellular Ca^2+^ as a result of continuous adrenergic activation induces cardiomyocyte apoptosis^30^, our results show that an excessive culture period at 42 °C induces cardiomyocyte apoptosis through TRPV-1-mediated Ca^2+^ overload, and the optimal duration of TRPV-1 activation might not be harmful for cardiomyocytes.

In the development of iPS cell-derived bioengineered tissues for regenerative medicine, it is important to reduce the contamination of residual iPS cells with less affecting tissue function. We previously reported that in the fabrication of cardiac cell sheets, a certain amount of fibroblasts is indispensable^31^,^32^. Consistent with our observation that 42 °C culture did not reduce the number of cardiac fibroblasts (Fig. 4c,d) or expression of extracellular matrixes, including Col1A and3A (Fig. 5a), when iPS cell-derived cardiac cells were transiently cultured at 42 °C, monolayered cardiac cell sheets were obtained after reducing the culture temperature (Fig. 5d). These findings indicate that 42 °C does not hinder bioengineered cardiac tissue fabrication. Cardiomyocytes are the major contractile elements in cardiac tissues, and the maintenance of cardiomyocyte function is indispensable for the fabrication of functional cardiac tissues. Even when cultured at 42 °C, the number of cTnT- and Nkx2.5-expressing cells (Fig. 4a,b), as well as mRNA expression of many cardiac contractile and secreting proteins, remained unchanged; TNNT2 expression significantly increased at 42 °C (Fig. 5a). These findings suggest that transcriptional and protein levels indicating cardiomyocyte function might be maintained even up to 2 days at 42 °C. Additionally we have demonstrated that beating cell sheets can still be fabricated after transient cultivation at 42 °C (Fig. 5d) and these cell sheets showed the spontaneous beating *in vivo* (Supplementary Video 10).

An estimation of remaining iPS cells is important for evaluating the risk of tumour formation following iPS cell-derived cell transplantation. Recently, Lin28 has been reported to be a high-sensitive marker gene for detecting residual iPS cells in tissues^5^. In the present study, when iPS cell-derived cardiac cells were cultured at 42 °C, Lin28 expression decreased in a time-dependent manner (Fig. 5b), while Oct4 expression remained unchanged (Fig. 5b), suggesting that Lin28 might be more sensitive for detecting elimination of residual iPS cells in cardiac tissues compared with Oct4. Furthermore, contamination of iPS cells in 1000 retinal pigment epithelial cells was detected as 0.1% Lin28 expression in iPS cells^5^, suggesting that Lin28 expression in iPS cells might be useful for estimating remaining iPS cells in tissues. In the present study, Lin28 expression levels by qPCR in iPS cell-derived cardiac cells after 42 °C, or OLDA treatment against iPS cells, were 0.4% (Fig. 7e). Moreover immunocytochemical analysis confirmed that very small percentage of Lin28-positive iPS cells (~0.1%) was detected after the cultivation at 42 °C for 2 days (Supplementary Fig. 4). Because Lin28 expression in human foetal and adult heart tissue was undetectable (data not shown), TRPV-1 activation strategies might eliminate remaining iPS cells in cardiac tissues, resulting in at most 0.4% iPS cell contamination.

It is also worth noting that although TRPV-1 activation strategies decreased the risk of contamination with remaining iPS cells, the expression of Lin28 was still slightly detected and very low number of Lin28-positive cells was observed in cardiac cell sheets tissues. Therefore we cannot exclude the possibilities of tumour formation upon transplantation.

Results from this study demonstrated that TRPV-1 activation via the combination of 42 °C culture and chemical compounds eliminated iPS cells in cardiac tissue, which may lead to reduced tumour formation following transplantation of iPS cell-derived cells. Recent studies have reported on an iPS cell elimination strategy using a methionine-free culture condition^6^,^7^ and human pluripotent stem cell-specific lectin-toxin fusion protein^8^. Tissue engineering technologies enable better engraftment of transplanted cells and subsequent functional improvement^33^, but undesirable residual iPS cells may also be engrafted, which may lead to tumour formation. The integration of these strategies with TRPV-1 activation strategies and developments of more efficient differentiation strategies will enable us to fabricate bioengineered tissues with low risk of tumour formation for regenerative medicine.

## Materials and Methods

### Antibodies and reagents

The following antibodies were used for immunocytochemistry: anti-cardiac troponin T (cTnT; Thermo Scientific, Rockford, IL, USA), anti-Vimentin (Abcam, Cambridge, UK) mouse monoclonal antibodies and anti-cardiac troponin T (Abcam), anti-Lin28 (Abcam) rabbit polyclonal antibody, and anti-Nkx2.5 (Santa Cruz Biotechnology Inc., Santa Cruz, CA, USA), and anti-Oct4 (R&D systems, Minneapolis, MN, USA) goat polyclonal antibodies. Secondary antibodies were purchased from Jackson ImmunoResearch Laboratories (West Grove, PA, USA). OLDA and Arvanil were purchased from Wako (Tokyo, Japan).

### Human iPSC culture

Human iPS cell lines (253G1, 201B7) were purchased from RIKEN (Tsukuba, Japan) and another cell line (1231A3) was kindly gifted from Kyoto University. For feeder culture experiments, iPS cells were maintained as described previously^32^ in Primate ES Cell Medium (ReproCELL, Yokohama, Japan) supplemented with 5 ng/ml basic fibroblast growth factor (ReproCELL) on mitomycin C-treated mouse embryonic fibroblasts (MEFs; ReproCELL) at 37 °C in humid air with 5% CO_2_. Cells were passaged as small clumps every 3–4 days using CTK solution (ReproCELL). For feeder-less culture experiments, iPS cells were adapted and maintained on iMatrix511 (Nippi, Tokyo, Japan) in StemFit AK03 (Ajinomoto, Tokyo, Japan). Cells were passaged as single cells every 7–8 days using TrypLE Select (Life Technologies, Carlsbad, CA, USA) as described elsewhere^34^. In some experiments, iPS cells were cultured at 40, 41, and 42 °C in humid air with 5% CO_2_. Sample images were obtained by an inverted microscopy (Nikon, Tokyo, Japan) with NIS-Elements software (Nikon).

### Preparation of hiPSCs expressing α-MHC promoter and rex-1 promoter-driven drug-resistance gene

A lentivirus vector (α-MHC-pure rex-1-neo) containing the puromycin-resistance gene under the control of the mouse α-myosin heavy chain (α-MHC) promoter and the neomycin-resistant gene under the control of the rex-1 promoter was purchased from Addgene (Cambridge, MA, USA) and used for preparing human iPS cells. Only undifferentiated cells express the recombinant gene by G418 (Life Technologies) for cell expansion and differentiated cardiomyocytes can be purified by puromycin treatment. The recombinant cell preparation was performed as follows:

- (i) Recombinant lentivirus preparationVector transfection and preparation of the recombinant lentivirus were performed by commercially available kits, Lipofectamine 2000 (Life Technologies) and ViraPower Lentiviral Packaging Mix (Life Technologies), respectively. Namely, HEK 293FT cells were seeded at 5 × 10^4^ cells/cm^2^ on a 100-mm polystyrene culture dish (Corning, Corning, NY, USA). After 24 h-cultivation, the vector (3 μg/dish) was transfected to the cells using Lipofectamine 2000 kit and OPTI-MEM (Life Technologies) for 8 h at 37 °C. Subsequently, medium containing vectors was removed; fresh medium was added and cells were further cultured. At 72 h after transfection, the culture supernatant containing recombinant lentivirus was harvested. The culture supernatant was centrifuged at 1700 × *g* for 5 min to eliminate large cell debris, then the supernatant was passed through a 0.45-μm filter (Merck Millipore, Billerica, MA, USA) to eliminate smaller cell debris. Then, the supernatant was concentrated 100-fold by ultracentrifugation using an ultracentrifuge (CP80β, Hitachi Koki, Tokyo, Japan) at 87,000 × *g* for 1.5 h at 4 °C, and concentrated virus was used to infect human iPS cells.
- (ii) Lentiviral infection protocolLentivirus infection was performed according to a previous report^35^ with a few modifications. Namely, human iPS cells (201B7), which were confluently cultured in one well of a 6-well plate (Corning), were dissociated with CTK solution. Small cell aggregations were resuspended in 1 mL of culture medium (Primate ES medium, ReproCELL) and transferred into a 15-ml centrifuge tube (Corning) for 5 min at room temperature. After 500 μL supernatant was removed, 400 μL fresh medium containing 8 μg Polybrene (Sigma-Aldrich, St. Louis, MO) was added. Finally, 100 μL concentrated virus supernatant was added and mixed with the cells and incubated at 37 °C for 6 h. The suspension was mixed occasionally during incubation, then seeded onto neomycin-resistant feeder cells, SL10 cells (ReproCELL), which were seeded onto two wells of a 6-well plate and cultured at 37 °C. After overnight incubation, 1 mL culture media was added to the cells and virus particles were washed out 36 hours after infection by a medium change. At 4 days after infection, iPS cells were treated with G418 sulfate (400 μg/mL) for 36 h. iPS cells were then rinsed twice with phosphate-buffered saline (PBS) to remove the drug, then the cells were cultured for several days. Retreatment with G418 sulfate was performed as necessary.

### Cardiac differentiation in the bioreactor and cardiac cell sheet preparation

The cardiac differentiation protocol in the bioreactor system (ABLE Co., Tokyo, Japan) has been described previously^32^. Prior to seeding cells, the surface of temperature-responsive dishes (UpCell; CellSeed, Tokyo, Japan) or cell culture plates (Corning) was coated with FBS for 2 h. After cardiac differentiation, the cells were dissociated with 0.05% trypsin/EDTA, cell aggregates were removed using a strainer (BD Biosciences, San Jose, CA, USA), and single cells were plated onto plates at 2.1 × 10^5^ cells/cm^2^ in DMEM (Sigma-Aldrich) supplemented with 10% FBS at 37 °C in humid air with 5% CO_2_. For some experiments, cardiomyocytes from iPS cells (201B7, αMHC-puro/Rex1-neo) were purified by treatment with puromycin (Sigma Aldrich, 1.5 μg/mL) for 1 day.

iPS-derived fibroblasts were obtained using a pre-plating technique. Following cardiac differentiation, cells were cultured onto non-coated culture plates (Corning) in DMEM supplemented with 10% FBS for 1 hour. The non-adherent cells were discarded and adherent cells were washed thoroughly three times with PBS and cultured in DMEM supplemented with 10% FBS. Over 99% of cells were positive for vimentin and the percentage of cTnT(+) cells was <1%. iPS cell-derived fibroblasts from passage 2–3 were used for the experiments.

### Co-culture experiment

Following cardiac differentiation of human iPS cells, cells were cultured for 2 days in 24-well culture plates (Corning) at a density of 2.1 × 10^5^ cells/cm^2^ in DMEM supplemented with 10% FBS at 37 °C in humid air with 5% CO_2_. One day before starting the co-culture experiment, iPS cells cultured on iMatrix511 were dissociated with TrypLE Select and a single cell suspension was cultured on EZ Sphere (AGC Techno Glass, Shizuoka, Japan) for 1 day in StemFit AK03 medium with Y27632 (10 μM) (Wako) to form cell aggregates. The following day, 50 cell aggregates of iPS cells were co-cultured with iPS-derived cardiac cells in 24-well culture plates in StemFit AK03 for 1 day. These preparations were then cultured for 2 days according to the following conditions: StemFit AK03 at 37 °C or 42 °C, 10% FBS DMEM at 37 °C or 42 °C.

### Immunocytochemistry

Cells were fixed with 4% paraformaldehyde, and immunocytochemistry was performed as described previously^32^. For the TUNEL assay, cells were stained using the Click-iT TUNEL Alexa Fluor 488 imaging assay kit (Life Technologies) according to manufacturer instructions. Nuclei were stained with Hoechst 33258 (Sigma-Aldrich). Samples were imaged by ImageXpress (Molecular Device, Sunnyvale, CA, USA) with MetaXpress and AcuityXpress software (Molecular Device).

### RNA extraction and quantitative RT-PCR

Total RNA extraction and RT-PCR were performed as described previously^7^. Primer pairs and Taqman MGB probes are shown in Table 1. Quantitative PCR was performed with a 7300 Real Time PCR System (Applied Biosystems, ABI). Relative mRNA expression levels were calculated using a standard curve of GAPDH or β-actin mRNA levels.

### TRPV-1 knockdown

iPS cells cultured on iMatrix511 were transfected with TRPV-1 siRNA (Life Technologies, 4392240) using Lipofectamine RNAi Max reagent (Life Technologies) according to manufacturer instructions. Silencer Select Negative Control siRNA (Life Technologies, 4390843) was used for the control.

### Live/Dead Staining

In some experiments, the viability of iPS cell-derived cardiomyocytes after the puromycin treatment or iPS cell-derived cells after the cardiac differentiation cultured at 37 °C or 42 °C were evaluated using Live/Dead staining kit (LIVE/DEAD^®^ Viability/Cytotoxicity Kit, Life Technologies) according to manufacturer instructions. Images were obtained by an inverted microscopy (Nikon) with NIS-Elements software (Nikon).

### Cell sheet transplantation

All animal experiments were performed according to the Guidelines of Tokyo Women’s Medical University on Animal Use, and consistent with the Guide for the Care and Use of Laboratory Animals prepared by the Institute of Laboratory Animal Resources (ILAR). All the experimental protocols were approved by the Institutional Animal Care and Use Committee of Tokyo Women’s Medical University. Two pairs of monolayered iPS cell-derived cardiac cell sheets that were cultured at 37 °C or 42 °C for 2 days (Fig. 5d) was collected by lowering culture temperature and 2 layered cardiac cell sheets from each condition were transplanted onto the subcutaneous tissue of a male fischer 344 athymic nude rat (aged 8 week) (Charles River Japan, Tokyo, Japan) as described previously^36^. One week after the transplantation, rats were were anaesthetized by 2% isoflurane inhalation and macroscopic images were recorded using surgical microscope system (Leica M651 Surgical microscope system, Germany).

### Statistical analysis

Data are presented as mean ± standard deviation. Statistical analyses were performed with the Student’s *t*-test for comparison between two groups. Multiple group comparisons were performed by one-way analysis of variance followed by Tukey-Kramer or Dunnett procedure for comparison of means. A value of *p* < 0.05 was considered statistically significant.

## Additional Information

**How to cite this article**: Matsuura, K. *et al*. TRPV-1-mediated elimination of residual iPS cells in bioengineered cardiac cell sheet tissues. *Sci. Rep.* **6**, 21747; doi: 10.1038/srep21747 (2016).

## Supplementary Material

Supplementary Information

Supplementary Video 1

Supplementary Video 2

Supplementary Video 3

Supplementary Video 4

Supplementary Video 5

Supplementary Video 6

Supplementary Video 7

Supplementary Video 8

Supplementary Video 9

Supplementary Video 10

Supplementary Video 11

Supplementary Video 12

Supplementary Video 13

## Figures and Tables

**Table 1:** Primers information.

Gene name	ABI No.
POU5F1 (OCT3/4)	Hs00999632_g1
Lin28	Hs00702808_s1
myosin, light chain 2, regulatory, cardiac, slow (MYL2)	Hs00166405_m1
myosin, light chain 7, regulatory (MYL7)	Hs01085598_g1
troponin T type 2 (cardiac) (TNNT2)	Hs00165960_m1
Collagen type I alpha 1 (COL1A1)	Hs00164004_m1
Collagen type III alpha1 (COL3A1)	Hs00943809_m1
natriuretic peptide A (NPPA)	Hs00383230_g1
natriuretic peptide B (NPPB)	Hs01057466_g1
TRPV-1	Hs00218912_m1
GAPDH	Hs00266705_g1
actin, beta	Hs99999903_m1
