# Supplementary material for: TRPV-1-mediated elimination of residual iPS cells in bioengineered cardiac cell sheet tissues
Source: Sci Rep. 2016 Feb 18;6:21747. doi: 10.1038/srep21747 (PMC4757885; doi:10.1038/srep21747)

TRPV-1-mediated elimination of residual iPS cells in bioengineered cardiac cell sheet tissues

Katsuhisa Matsuura[1, 2]*, Hiroyoshi Seta[1, 3], Yuji Haraguchi[1], Khaled Alsayegh[1], Hidekazu Sekine[1], Tatsuya Shimizu[1], Nobuhisa Hagiwara[2], Kenji Yamazaki[3], Teruo Okano[1]

Supplementary figure legends

Supplementary Figure 1. Number of Oct4(+) cells of feeder-less iPS cells in 42 °C culture. (a) Human iPS cells cultured on laminin E8 fragment were cultured at 37 °C or 42 °C for 1 and 2 days, respectively. Upper panels are representative of phase-contrast images. Bars, 100 µm. Middle panels are representative images of Oct4 (green) and Hoechst (blue) staining. Bars, 200 µm. Lower, montage images of 36 fields (6 × 6) of Oct4 staining images (original magnification of each field is ×20). (b) The Oct4(+) cell number in 36 fields was calculated and shown in the graph (n = 3). *p < 0.01 vs. day 0.

Supplementary Figure 2. Influence of 42 °C culture on 1231A3 Ff-iPS cells. (a) 1231A3 episomal Ff-iPS cells were cultured at 37 °C or 42 °C for 2 days. Upper panels are representative of phase-contrast images. Bars, 100 µm. Middle panels are representative images of Oct4 (green) and Hoechst (blue) staining. Bars, 200 µm. Lower, montage images of 36 fields (6 × 6) of Oct4 staining images (original magnification of each field is ×20). (b) Total (left) and Oct4(+) (right) cell number in 36 fields was calculated and shown in the graph (n = 3).

Supplementary Figure 3. Live/Dead staining analysis. (a) iPS cell-derived purified cardiomyocytes were cultured at 37 °C or 42 °C for 1 or 2 days. Panels are representative images of Live (green) and Dead (red) cells. Bars, 100 µm. (b) iPS cell-derived non-purified cardiac cells were cultured at 37 °C or 42 °C for 1 or 2 days. Panels are representative images of Live (green) and Dead (red) cells. Bars, 100 µm.

Supplementary Figure 4. Lin28 expressing cells in cardiac cell sheet tissues. (a) iPS cell-derived cardiac cells were cultured at 37 °C or 42 °C for 2 days. (a) Panels are representative images of Lin28 (green) and Hoechst (blue) staining. Bars, 200 µm. Arrows indicate Lin28 expressing cells. iPS cells (left panels) were used as positive

control for Lin28 staining. (b) The percentage of Lin28 positive cells were calculated and shown in the graph (n = 4).

Supplementary Figure 5. iPS elimination using TRPV-1 agonist. (a and b) iPS cells on laminin E8 fragment were cultured with Arvanil for 1 day. (a) Upper panels are representative of phase-contrast images. Bars, 100 µm. Middle panels, Hoechst staining images. Bars, 200 µm. Lower, montage images of 36 fields (6 × 6) of Hoechst staining images (original magnification of each field is ×20). (b) Cell number at each condition was calculated and shown in the graph (n = 3).

Supplementary Video 1. Spontaneous beating of iPS cell-derived cardiomyocytes cultured at 37 °C for 1 day. The original magnification is ×10.

Supplementary Video 2. Spontaneous beating of iPS cell-derived cardiomyocytes cultured at 37 °C for 2 days. The original magnification is ×10.

Supplementary Video 3. Spontaneous beating of iPS cell-derived cardiomyocytes

cultured at 42 °C for 1 day. The original magnification is ×10.

Supplementary Video 4. Spontaneous beating of iPS cell-derived cardiomyocytes cultured at 42 °C for 2 days. The original magnification is ×10.

Supplementary Video 5. Spontaneous beating of iPS cell-derived cardiomyocytes 1 week after the transient culture at 37 °C for 2 days. The original magnification is ×10.

Supplementary Video 6 Spontaneous beating of iPS cell-derived cardiomyocytes 1 week after the transient culture at 42 °C for 2 days. The original magnification is ×10.

Supplementary Video 7. Spontaneous beating of iPS cell-derived cardiac cell sheet preparation. Cells were cultured for 4 days at 37 °C. The original magnification is ×10.

Supplementary Video 8. Spontaneous beating of iPS cell-derived cardiac cell sheet preparation. Cells were cultured at 37 °C from days 0 to 1 and days 3 to 4 and at 42 °C from days 1 to 3. The original magnification is ×10.

Supplementary Video 9. Spontaneous beating of iPS cell-derived cardiac cell sheet tissues in vivo. Two layered of cardiac cell sheets that were cultured at 37 °C for 2 days were transplanted onto the subcutaneous tissue of nude rats. The movie was recorded 1 week after the transplantation.

Supplementary Video 10. Spontaneous beating of iPS cell-derived cardiac cell sheet tissues in vivo. Two layered of cardiac cell sheets that were cultured at 42 °C for 2 days were transplanted onto the subcutaneous tissue of nude rats. The movie was recorded 1 week after the transplantation.

Supplementary Video 11. Spontaneous beating of iPS cell-derived cardiomyocytes cultured with vehicle for 1 day. The original magnification is ×10.

Supplementary Video 12. Spontaneous beating of iPS cell-derived cardiomyocytes cultured with OLDA (5 μM) for 1 day. The original magnification is ×10.

Supplementary Video 13. Spontaneous beating of iPS cell-derived cardiomyocytes cultured with OLDA (0.5 μM) for 1 day. The original magnification is ×10.

Supplementary Figure 1

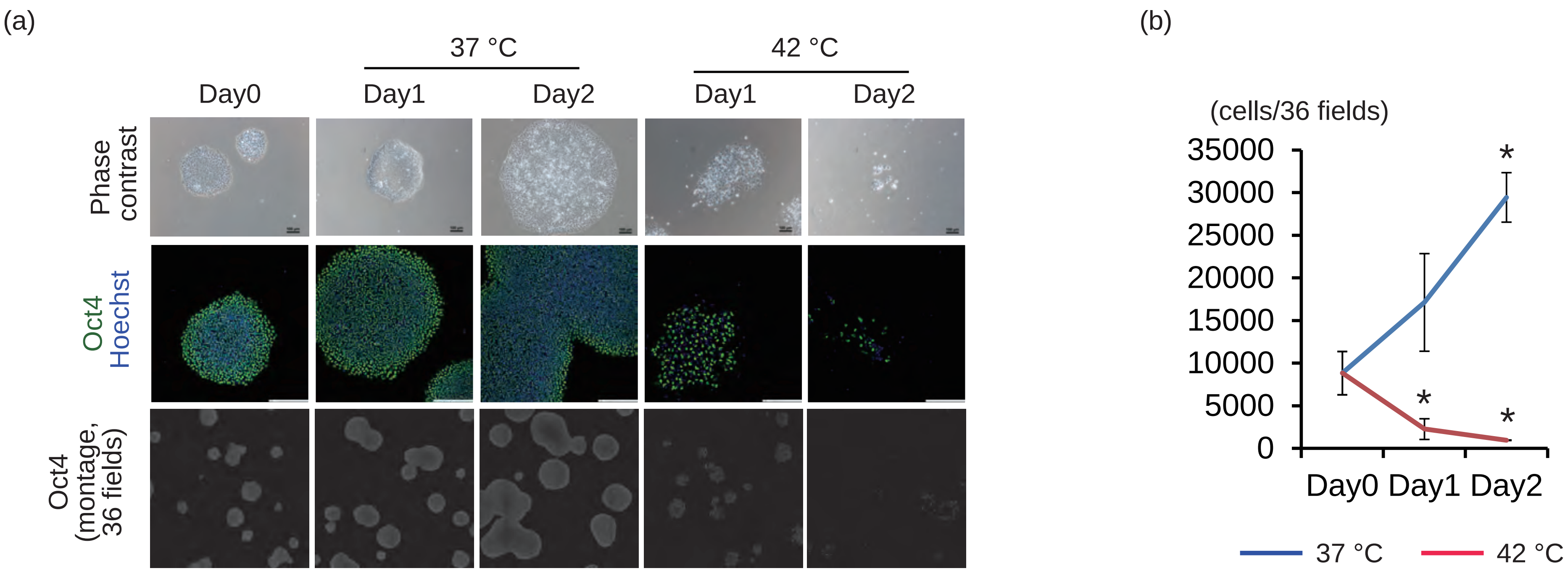

Supplementary Figure 2

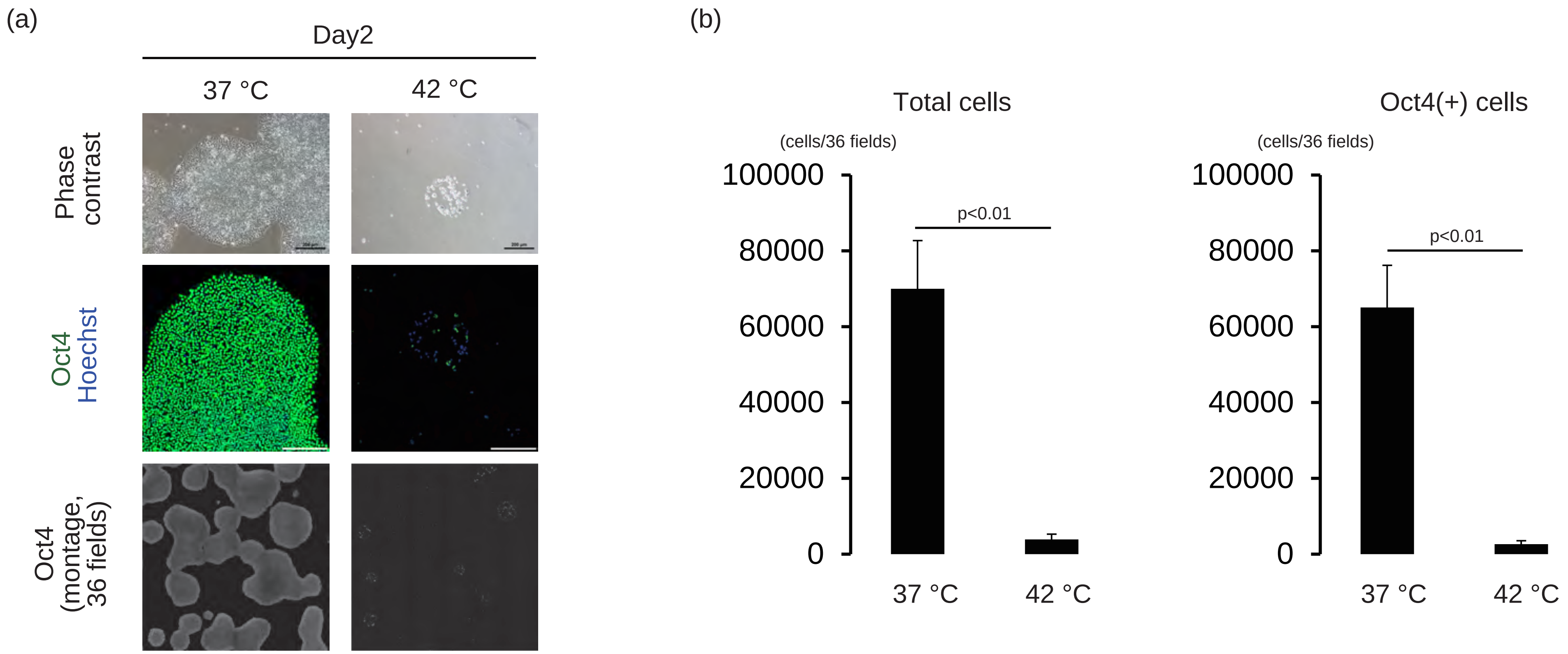

Supplementary Figure 3

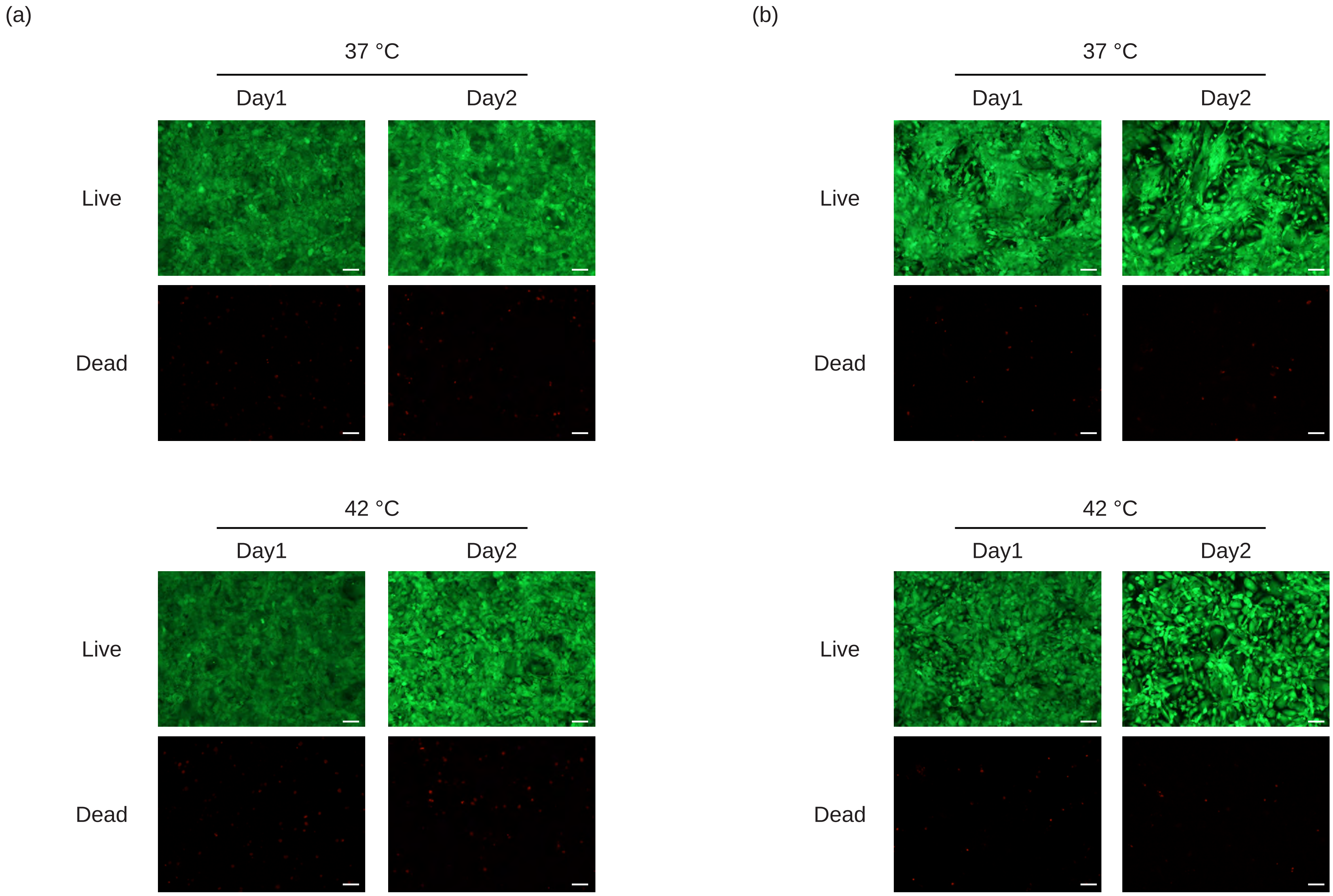

Supplementary Figure 4

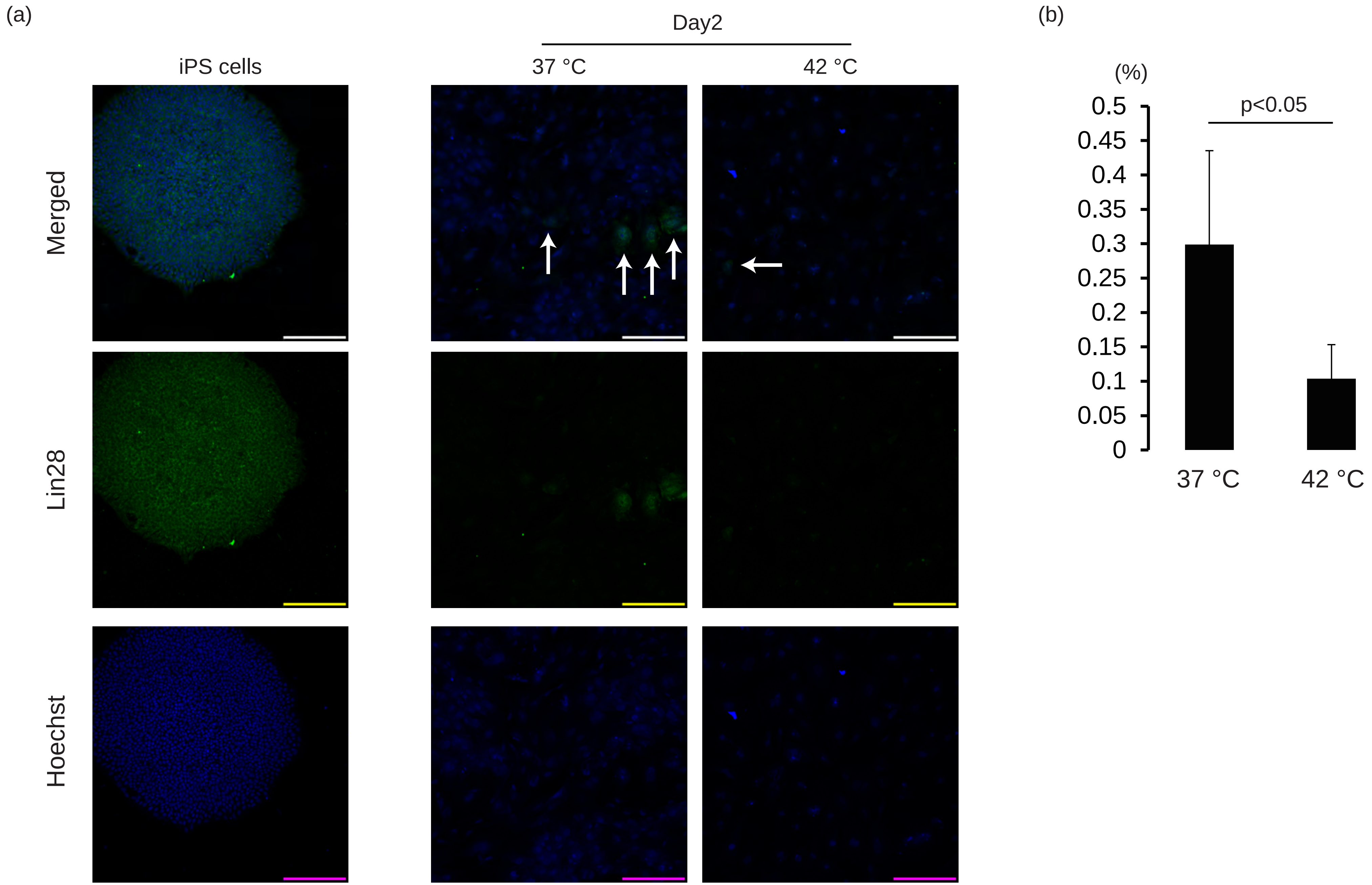

Supplementary Figure 5

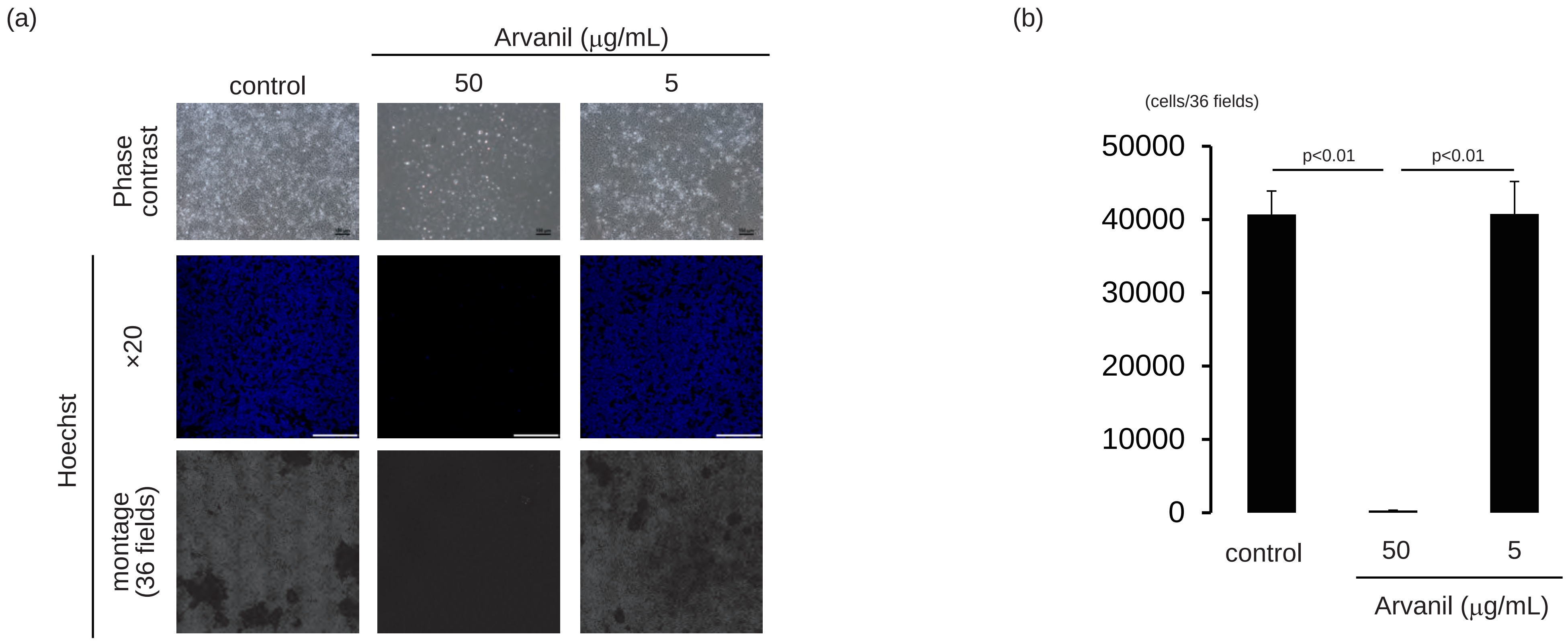

Supplement: Supplementary Information [file srep21747-s1.pdf]
